# Modeling and Analysis of the Molecular Basis of Pain in Sensory Neurons

**DOI:** 10.1371/journal.pone.0006758

**Published:** 2009-09-11

**Authors:** Sang Ok Song, Jeffrey Varner

**Affiliations:** School of Chemical and Biomolecular Engineering, Cornell University, Ithaca, New York, United States of America; Virginia Tech, United States of America

## Abstract

Intracellular calcium dynamics are critical to cellular functions like pain transmission. Extracellular ATP plays an important role in modulating intracellular calcium levels by interacting with the P2 family of surface receptors. In this study, we developed a mechanistic mathematical model of ATP-induced P2 mediated calcium signaling in archetype sensory neurons. The model architecture, which described 90 species connected by 162 interactions, was formulated by aggregating disparate molecular modules from literature. Unlike previous models, only mass action kinetics were used to describe the rate of molecular interactions. Thus, the majority of the 252 unknown model parameters were either association, dissociation or catalytic rate constants. Model parameters were estimated from nine independent data sets taken from multiple laboratories. The training data consisted of both dynamic and steady-state measurements. However, because of the complexity of the calcium network, we were unable to estimate unique model parameters. Instead, we estimated a family or *ensemble* of probable parameter sets using a multi-objective thermal ensemble method. Each member of the ensemble met an error criterion and was located along or near the optimal trade-off surface between the individual training data sets. The model quantitatively reproduced experimental measurements from dorsal root ganglion neurons as a function of extracellular ATP forcing. Hypothesized architecture linking phosphoinositide regulation with P2X receptor activity explained the inhibition of P2X-mediated current flow by activated metabotropic P2Y receptors. Sensitivity analysis using individual and the whole system outputs suggested which molecular subsystems were most important following P2 activation. Taken together, modeling and analysis of ATP-induced P2 mediated calcium signaling generated qualitative insight into the critical interactions controlling ATP induced calcium dynamics. Understanding these critical interactions may prove useful for the design of the next generation of molecular pain management strategies.

## Introduction

Millions worldwide suffer daily from acute and chronic pain. Extracellular ATP plays an important role in pain transduction in both the periphery and central nervous systems. ATP released from damaged tissue can activate sensory receptors (nociceptors) and contribute to increased pain sensitivity [1]. Subcutaneous administration of ATP or its analog 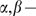 methylene ATP (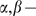 meATP) has been linked with pain in animals and humans [2]–[5]. ATP initiates pain by interacting with the P2 family of surface receptors. P2 receptors can be divided into ionotropic P2X receptors (ligand-gated ion channels) and metabotropic P2Y Gq-protein coupled receptors. This classification is based on molecular structure and signal transduction mechanism [6], [7]. Activated P2 receptors are either directly (P2X) or indirectly (P2Y) responsible for the transport of calcium into the cytosol. Intracellular calcium levels are important in several neuronal functions like transmitter release, membrane excitability and protein/gene regulation [8]–[13]. Calcium levels are also important in cell proliferation, differentiation, and death programs [14].

P2 receptors have been implicated in pain transmission in the peripheral and central nervous systems. Different P2X receptor subtypes e.g., P2X3 and P2X2/3 are localized on capsicaicin-sensitive, isolectin B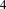 (IB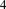) binding, small-sized Dorsal Root Ganglion (DRG) neurons [15], [16]. These receptors are involved in several pain states including migraine headaches [17]–[22]. ATP activates P2X receptors by binding, leading to slowly (P2X2/3) and rapidly (P2X3) desensitizing transmembrane currents [23]. Conversely, P2Y receptors transduce signals through a Gq-coupled protein cascade leading to IP3-IP3R channel activation [7]. P2Y2 receptors are equipotently activated by both ATP and UTP in a variety of cell types [7], [24]–[26]. Eight different P2Y receptors have been identified in humans [7]. P2Y1 and P2Y2 receptors are highly expressed in small DRG sensory neurons [27], medium and large-size sensory neurons [24], [28], [29] and linked with action potential in afferent nerve fibers [30], [31]. However, their role in P2X regulation or the transmission of pain signals remains unclear.

## Results

**Figure 1:**
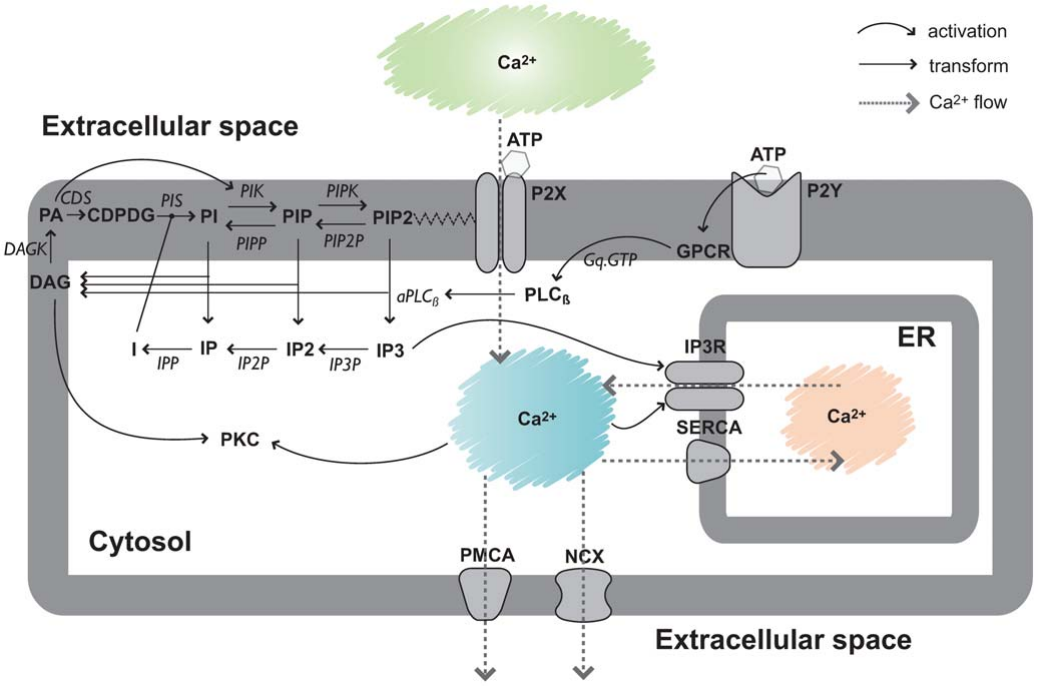
Schematic of calcium signaling network used in this study. Ca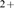 can enter the cytosol via P2X channels, inositol trisphosphate receptors (IP3R) and passive Ca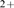 leakage. ATP binding to P2X activates the channel and induces a rapid increase in cytosolic Ca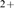 in the presence of extracellular calcium. ATP binding to P2Y receptors activates membrane-bound phospholipase C (PLC) which hydrolyzes phosphatidylinositol-4, 5-bisphophate (PIP2) into inositol 1,4,5-trisphosphate (IP3) and diacylglycerol (DAG). Cytosolic calcium and IP3 binding triggers the opening of IP3R channels and the subsequent release of endogenous Ca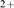 from the Endoplasmic Reticulum (ER) into the cytosol. Cytosolic Ca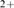 is translocated to the extracellular medium by plasma membrane Ca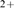 ATPase (PMCA) pumps, Na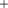/Ca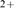 exchangers (NCX) and to the ER by Sarcoplasmic/Endoplasmic Reticulum Ca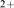 (SERCA) ATPase pumps. Phosphoinositides (PIs) are recycled between the plasma membrane and cytosol by phosphorylation and dephosphorylation events. The specific reactions, kinetic constants and non-zero initial conditions used in this study are given in Table 1 and Table 2, respectively.

In this study, we developed a mechanistic mathematical model of P2 driven calcium signaling in archetype sensory neurons. The model architecture, which described 90 species connected by 162 interactions, was formulated by aggregating disparate molecular modules from literature [32]–[35]. While the interaction network was similar (but not identical) to these previous studies, we used a different modeling strategy to describe the kinetics and identify the model parameters. The model described P2Y/P2X surface receptor activation (including Gq protein signaling), Phophoinositide (PI) metabolism, ATPase pumps, Na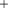/Ca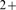 exchangers, ion leaks and IP3R channels (Fig. 1 and Table 1). We used only elementary mass-action kinetics to describe the rate of each molecular interaction. The mass-action formulation, while expanding the dimension of the P2 calcium model, regularized the mathematical structure. For example, each model interaction was associated with a single parameter. The regular structure also allowed automatic generation of the model equations and components required for model analysis. Mass-action kinetics also regularized the model parameters. Unknown model parameters were one of only three types, association, dissociation or catalytic rate constants. Thus, although mass-action kinetics increased the number of parameters and species, they reduced the complexity of model analysis. The one exception was the kinetics of flow through gated channels which was parameterized by permeability constants and modeled using the Nernst equation. In addition, while we assumed spatial homogeneity in any single compartment, we differentiated between cytosolic, Endoplasmic Reticulum (ER) and membrane localized species and processes. The model had 252 unknown parameters (initial conditions and kinetic constants, Table 2). Model parameters were estimated from nine independent data sets taken from multiple laboratories and different cell-lines (Table 3). The training data consisted of both dynamic and steady-state measurements. However, we were unable to estimate unique model parameters from the training data. Instead, we estimated a family or *ensemble* of probable model parameter sets [36]–[38] using a Multi-Objective Thermal Ensemble (MOTE) technique (materials and methods). Each member of the ensemble met a training error criterion and was located along or near the optimal trade-off surface between the individual training data sets. Thus, while we did not uniquely determine the model parameters, we constrained their values to regions that were consistent with observations. Sensitivity analysis was then conducted over the parameter ensemble to better understand the role and importance of the model parameters. All model code as well as all code used in the parameter identification studies is available in the supplemental materials (Supplemental Materials S1).

**Table 1:** Reactions and parameter values used in this study.

Reaction	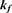	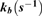	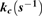	Source
**P2X3 receptor**				
ATPx+P2X3 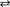 ATP-P2X3	0.95 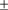 1.27	0.64 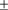 3.97	-	[33]
ATPx+ATP-P2X3 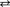 ATP2-P2X3	5.19 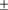 3.40	0.76 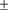 4.86	-	[33]
ATPx+ATP2-P2X3 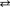 ATP3-P2X3	0.56 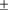 0.66	1.97 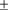 10.5	-	[33]
ATP3-P2X3 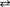 P2X3open	154 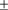 96.5	0.03 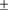 0.15	-	[33]
P2X3 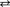 P2X3d	2.1e-6 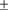 1.8e-6	0.02 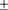 0.07	-	[33]
ATP-P2X3 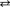 ATP-P2X3d	2.15 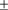 3.13	3.7e-6 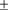 3.0e-6	-	[33]
ATP2-P2X3 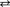 ATP2-P2X3d	1.9e-6 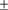 1.5e-6	2.5e-6 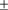 3.2e-6	-	[33]
ATP3-P2X3 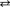 ATP3-P2X3d	2.5e-6 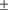 3.2e-6	2.3e-6 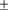 1.9e-6	-	[33]
P2X3open 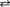 ATP3-P2X3df	0.91 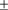 1.19	4.2e-4 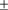 5.3e-4	-	[33]
ATPx+P2X3d 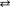 ATP-P2X3d	0.56 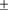 3.33	0.007 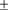 0.008	-	[33]
ATPx+ATP-P2X3d 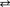 ATP2-P2X3d	2.61 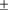 3.58	0.007 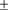 0.007	-	[33]
ATPx+ATP2-P2X3d 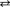 ATP3-P2X3d	1.53 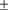 2.35	0.006 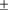 0.006	-	[33]
ATP3-P2X3d 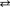 ATP3-P2X3df	2.6e-5 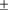 3.4e-5	0.003 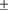 0.018	-	[33]
P2X3open+n*PIP2 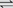 PIP2-P2X3open	0.006 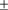 0.011	0.10 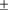 0.17	-	-
PIP2-P2X3open 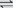 ATP3-P2X3df+n*PIP2	0.097 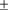 0.095	3.7e-6 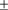 2.4e-6	-	-
**P2Y receptor and G-protein cascade**				
ATPx+P2Y 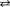 ATP-P2Y	0.12 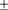 0.66	47.2 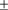 141	-	[24], [92]
ATP-P2Y 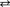 ATP-P2Yact	2.86 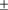 3.66	4.58 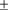 5.73	-	[92]
ATP-P2Yact+Gq-GDP 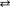 ATP-P2Y-Gq	12.6 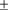 9.78	70.2 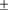 79.4	-	[50]
ATP-P2Y-Gq 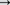 Gq 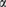 -GTP+G 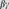 +ATP-P2Y	-	-	0.70 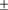 0.68	[50]
Gq-GDP 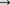 Gq 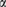 -GTP+G 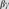	-	-	4.7e-6 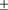 1.8e-5	[32]
Gq 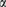 -GTP 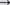 Gq 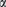 -GDP	-	-	0.001 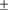 0.003	[93]
Gq 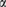 -GDP+G 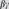 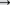 Gq-GDP	-	-	0.24 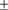 1.49	[32]
**PLC** 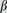 ** cascade and IP3/DAG generation**				
PLC 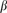 +CAi 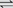 PLC 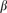 -Ca	2.00 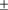 4.60	0.30 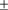 1.18	-	[94], [95]
PLC 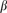 +Gq 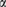 -GTP 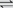 PLC 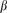 -Gq	0.25 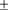 0.34	0.03 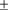 0.19	-	[94], [95]
PLC 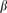 -Ca+Gq 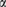 -GTP 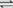 PLC 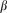 -Ca-Gq	262 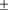 248	0.004 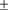 0.02	-	[94], [95]
PLC 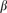 -Gq+CAi 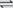 PLC 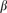 -Ca-Gq	14.1 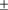 18.4	0.016 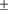 0.01	-	[94], [95]
PLC 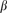 -Gq 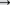 PLC 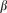 +Gq 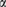 -GDP	-	-	0.094 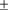 0.047	[94], [95]
PLC 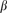 -Ca-Gq 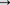 PLC 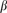 -Ca+Gq 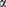 -GDP	-	-	1.79 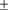 1.75	[94], [95]
PLC 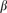 -Ca-Gq+PIP2 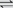 PLC 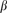 -Ca-Gq-PIP2	226 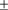 223	32.3 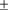 35.3	-	[94], [95]
PLC 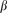 -Ca+PIP2 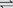 PLC 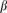 -Ca-PIP2	1.3e-4 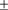 2.3e-4	0.002 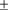 0.008	-	[94], [95]
PLC 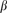 -Ca-PIP2 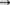 PLC 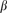 -Ca+IP3+DAG	-	-	2.5e-4 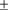 1.5e-3	[94], [95]
PLC 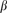 -Ca-Gq-PIP2 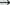 PLC 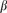 -Ca-Gq+IP3+DAG	-	-	587 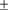 370	[94], [95]
PLC 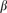 -Ca-Gq+PIP 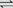 PLC 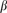 -Ca-Gq-PIP	0.72 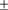 4.38	11.7 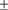 16.6	-	[94], [95]
PLC 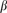 -Ca+PIP 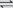 PLC 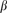 -Ca-PIP	3.2e-5 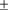 1.9e-4	0.003 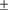 0.006	-	[94], [95]
PLC 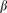 -Ca-PIP 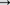 PLC 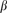 -Ca+IP2+DAG	-	-	4.8e-4 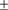 1.1e-3	[94], [95]
PLC 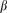 -Ca-Gq-PIP 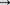 PLC 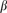 -Ca-Gq+IP2+DAG	-	-	2.7 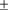 3.9	[94], [95]
PLC 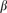 -Ca-Gq+PI 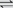 PLC 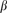 -Ca-Gq-PI	0.25 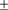 1.56	92.5 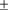 139	-	[94], [95]
PLC 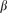 -Ca+PI 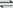 PLC 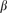 -Ca-PI	4.1e-5 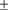 1.7e-4	0.003 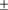 0.006	-	[94], [95]
PLC 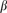 -Ca-PI 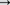 PLC 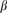 -Ca+IP+DAG	-	-	7.1e-4 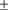 1.3e-3	[94], [95]
PLC 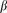 -Ca-Gq-PI 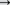 PLC 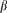 -Ca-Gq+IP+DAG	-	-	0.75 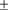 2.83	[94], [95]
PKC+DAG 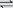 PKC-DAG	0.22 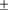 1.13	0.03 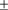 0.15	-	-
PKC+CAi 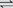 PKC-CA	6.4e-4 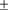 7.0e-4	0.61 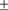 3.05	-	-
PKC-DAG+CAi 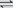 PKC-DAG-CA	9.99 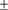 15.8	20.7 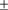 32.2	-	-
PKC-CA+DAG 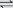 PKC-DAG-CA	3.21 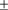 16.2	3.6e-4 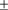 1.4e-3	-	-
PKC-DAG-CA 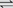 PKCa	0.06 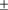 0.25	0.001 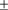 0.001	-	-
PKCa+PLC 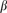 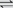 PKCa-PLC 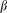	0.29 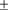 1.67	0.89 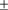 3.77	-	-
PKCa-PLC 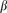 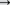 PKCa+pPLC 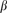	-	-	1.43 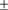 2.10	-
pPLC 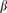 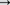 PLC 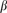	-	-	0.006 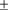 0.02	-
PKCa+PLC 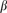 -Ca 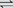 PKCa-PLC 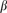 -Ca	66.7 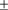 103	14.1 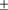 12.4	-	-
PKCa-PLC 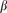 -Ca 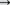 PKCa+pPLC 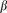 -Ca	-	-	0.50 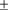 1.63	-
pPLC 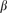 -Ca 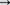 PLC 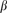 -Ca	-	-	0.005 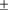 0.022	-
**PI signaling**				
PI+PIK 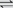 PI-PIK	957 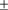 813	146 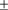 133	-	[34]
PI-PIK 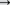 PIK+PIP	-	-	78.8 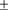 32.2	[34]
PIP+PIPP 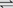 PIP-PIPP	1741 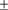 918	152 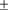 126	-	–
PIP-PIPP 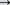 PI+PIPP	-	-	61.7 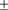 34.5	–
PIP+PIPK 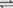 PIP-PIPK	243 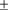 203	949 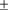 717	-	[34]
PIP-PIPK 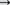 PIPK+PIP2	-	-	163 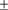 87.0	[34]
PIP+PA-PIPK 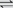 PIP-PA-PIPK	329 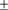 212	837 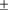 1276	-	–
PIP-PA-PIPK 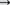 PA-PIPK+PIP2	-	-	60.1 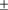 33.5	–
PIP2+PIP2P 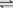 PIP2-PIP2P	346 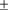 211	1212 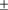 1741	-	–
PIP2-PIP2P 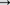 PIP+PIP2P	-	-	145 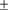 119	–
IP3+IP3P 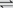 IP3-IP3P	117 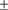 52.8	533 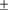 188	-	[34]
IP3-IP3P 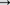 IP2+IP3P	-	-	109 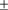 63.1	[34]
IP2+IP2P 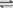 IP2-IP2P	78.9 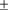 46.1	94.7 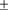 69.0	-	[34]
IP2-IP2P 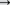 IP+IP2P	-	-	44.0 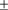 28.5	[34]
IP+IPP 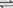 IP-IPP	849 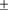 894	245 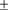 140	-	[34]
IP-IPP 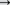 Ins+IPP	-	-	37.5 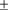 19.6	[34]
DAG+DAGK 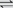 DAG-DAGK	250 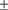 111	86.5 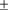 59.1	-	[34]
DAG-DAGK 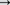 PA+DAGK	-	-	62.9 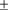 36.2	[34]
PA+CDS 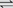 PA-CDS	22.4 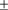 12.7	110 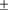 61.6	-	[34]
PA-CDS 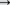 CDPDG+CDS	-	-	17.7 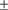 14.5	[34]
CDPDG+Ins 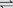 CDPDG-Ins	33.1 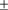 27.5	51.5 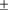 46.1	-	[34]
CDPDG-Ins+PIS 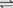 CDPDG-Ins-PIS	26.9 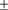 37.8	104.7 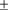 87.3	-	[34]
CDPDG-Ins-PIS 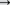 PI+PIS	-	-	36.6 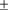 23.2	[34]
PA+PIPK 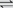 PA-PIPK	178 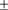 75.5	6.80 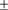 4.07	-	[34]
**IP3R channel**				
IP3R+IP3 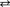 IP3R-IP3	1.20 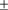 1.93	300 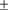 128	-	[42], [46], [47]
IP3R-IP3 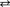 IP3R-IP3a	1550 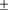 516	63.7 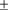 54.1	-	[42], [46], [47]
IP3R-IP3a+Ca 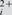 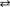 IP3R-IP3-CA	65.3 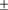 34.1	71.7 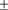 42.8	-	[42], [46], [47]
IP3R-IP3-CA 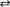 IP3Ropen	409 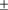 315	47.3 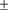 32.5	-	[42], [46], [47]
IP3R-IP3-CA+Ca 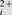 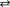 IP3R-IP3-2CA	3.58 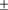 1.03	0.36 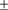 0.20	-	[42], [46], [47]
IP3R-IP3-2CA 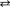 IP3Ri	0.52 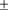 0.10	0.03 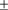 0.02	-	[42], [46], [47]
IP3R-IP3-2CA+Ca 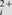 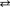 IP3R-IP3-3CA	3.07 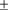 2.29	0.95 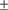 0.56	-	[42], [46], [47]
IP3R-IP3-3CA 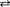 IP3Rii	0.10 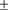 0.08	0.04 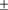 0.016	-	[42], [46], [47]
**Ca** 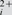 ** permeation through channels and leaks**				
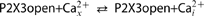	0.98 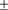 0.52	0.98 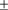 0.52	-	[33], [56]
PIP2-IP3Ropen+Ca 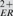 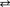 PIP2-IP3Ropen+Ca 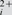	4.52 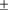 1.83	4.52 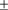 1.83	-	[24], [42], [46], [47]
IP3Ropen+Ca 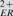 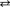 IP3Ropen+Ca 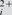	14.2 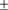 6.51	14.2 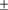 6.51	-	[24], [42], [46], [47]
Ca 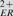 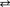 Ca 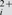	5.3e-4 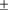 5.3e-4	5.3e-4 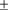 5.3e-4	-	-
Ca 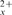 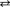 Ca 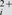	2.2e-5 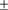 1.4e-5	2.2e-5 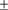 1.4e-5	-	-
**Ca** 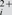 ** pumps and exchangers**				
SERCA+2Ca 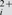 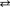 SERCA-2Ca	2969 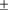 1666	87.0 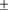 69.7	-	[96]
SERCA-2Ca 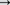 SERCA+2Ca 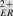	-	-	208 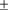 83.8	[96]
PMCA+Ca 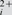 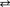 PMCA-Ca	59.6 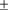 43.7	289 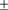 173	-	[97]
PMCA-Ca 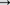 PMCA+Ca 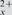	-	-	26.3 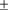 15.3	[97]
NCX+2Ca 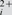 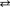 NCX-2Ca	4.60 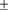 7.38	1630 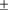 589	-	[97]
NCX-2Ca 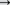 NCX+2Ca 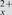	-	-	59.6 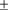 24.2	[97]

Values for the kinetic parameters and network structure were taken from the literature or estimated from experimental data. The kinetics of binding and catalytic interactions were assumed to follow mass-action rate laws. The quantity 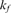 denotes forward rate constants, 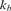 denotes backward rate constants and 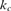 denotes catalytic rate constants. All binding interactions were assumed to be reversible. Unless otherwise specified, zero-order rate constants had units of 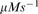, first-order rate constants had units of 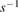, and second-order rate constants had units of 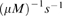. The mean and standard deviation over the parameter ensemble are reported for each kinetic parameter. The value of the P2X3 and IP3R channel permeability constants have no direct literature sources and were estimated separately from data [24], [33], [56]. Leakage constants were adjusted so that the mean steady-state cytosolic calcium concentration without agonist was 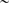
0.05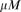.

**Table 2:** Non-zero initial conditions estimated in this study.

Initial Value	Species
0.46 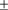 0.21	P2X3
8.42 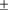 4.62	PIP2
0.31 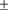 0.18	P2Y
3.50 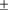 1.62	Gq-GDP
0.14 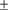 0.06	PLCb
0.06 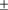 0.04	CAi
0.13 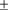 0.06	IP3
7.54 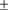 4.54	PIP
10.6 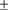 3.83	PI
0.09 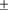 0.02	PKC
0.18 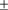 0.07	PIK
0.091 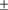 0.040	PIPP
0.046 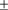 0.027	PIPK
0.057 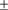 0.038	PIP2P
0.013 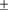 0.011	IP3P
0.03 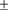 0.01	IP2P
0.005 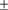 0.002	IPP
0.16 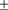 0.20	DAGK
0.16 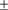 0.10	CDS
0.01 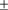 0.006	PIS
0.27 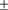 0.08	IP3R
1634 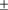 997.0	CAx
90.8 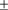 116.0	CAs
0.014 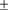 0.01	SERCA
0.096 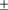 0.009	PMCA
0.026 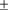 0.009	NCX

Unless otherwise specified, all concentrations had units of 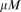. The mean and standard over the parameter ensemble are reported.

**Table 3:** Experimental training data used to estimate the ensemble of the model parameters (Fig. 3).

	Observation	Stimulation	Cell line	Source
A	gated IP3R fraction	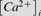 dependent	ER vesicles from canine cerebellum	[47]
B	gated IP3R fraction	[IP3] dependent	ER vesicles from canine cerebellum	[46]
C	[PIP] transient	GPCR activation	SH-SY5Y cells	[49]
D	[PIP2] transient	GPCR activation	SH-SY5Y cells	[49]
E	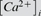 transient	100 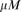 M ATP	P2X3-transfected GT1 cells	[56]
F	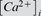 transient	100 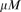 M ATP	Neuro2a cells	[48]
G	P2X3 current peak	ATP-dose dependent	rat DRG neurons	[31]
H	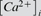 peak	ATP-dose dependent	rat DRG neurons	[24]

### Independent training sets constrained the behavior of the model

**Figure 2:**
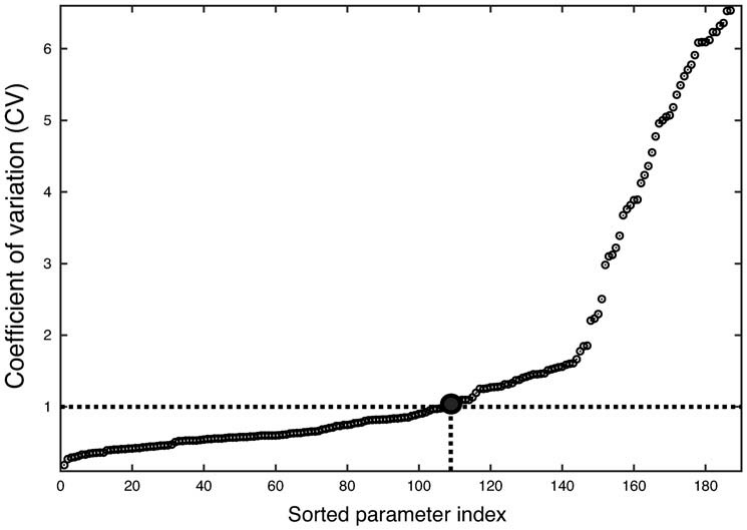
Coefficient of Variation (CV) of parameters (reaction rate constants and non-zero initial conditions) in the ensemble. Thirty-one parameters were constrained with a CV of less than or equal to 0.5 and 108 had a CV of less than one. The minimum CV was 0.18 while the maximum was 6.5.

Models of signal transduction networks often exhibit complex relationships between model performance and parameter values [38]. It is rarely possible to uniquely identify parameters from noisy experimental measurements, even when given extensive training data [39]. Uncertainty in model parameters translates to uncertainty in model simulations. To address uncertainty in the calcium model parameters, we estimated a family of possible parameter sets using a MOTE technique. The 252 unknown parameters (initial conditions and kinetic constants) were estimated using nine data sets from multiple laboratories. Training data was selected to approximately constrain the behavior of each of the submodels in the integrated model. Because the training data consisted of both steady-state and time-series measurements taken from multiple sources, it contained intrinsic conflicts. To balance these conflicts, we treated each training set as a separate objective in a multiple objective optimization calculation. Parameter values were adjusted to minimize the squared error between model simulations and experimental measurements. We generated 250 parameters sets on or near the Pareto-optimal frontier and finally selected 123 parameters just on the Pareto-optimal frontier. The number of parameter sets obtained was constrained by computational demands. The ensemble reported here required greater than 20,000 annealing runs and 107 hours on an Apple 2.6 Ghz Intel Core 2 Duo workstation (Apple Computer, Cupertino CA). In the ensemble, 31 parameters had a Coefficient of Variation (CV) of less than 0.5 while 108 had a CV of less than one. The minimum CV was 0.18 while the maximum was 6.5. The most constrained parameters were largely associated with IP3R regulation while the dissociation rates of PLC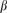-Ca-Gq complex or ATP-P2X3R complexes were least constrained. Most of non-zero initial conditions (92%) had a CV of less than one (Fig. 2).

**Figure 3:**
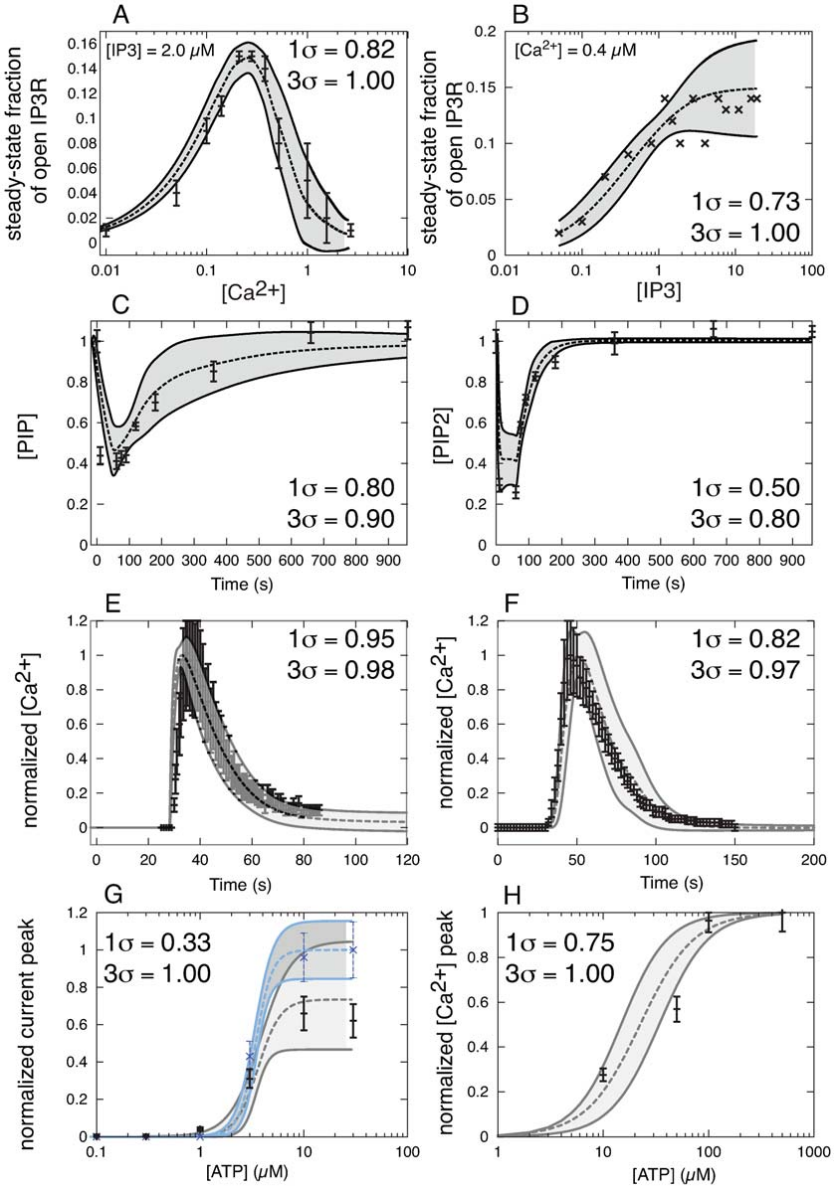
Comparison of model simulations versus training data. The dashed lines in each case denote the mean simulated value over the ensemble of model parameters while the shaded regions denote one ensemble standard deviation (N = 123). Experimental data are shown with error bars. In each corner, the fraction of experimental points captured at one and three standard deviations is given. (A,B): Steady state fraction of open IP3R channels as a function of cytosolic Ca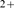 (A) and IP3 concentration (B). The experimental data was reproduced from Bezprozvanny *et al.* [47] and Watras *et al.* [46], respectively. (C,D): Time-resolved measurements of PIP (C) and PIP2 (D) levels following GPCR activation in SH-SY5Y cells. The PIP/PIP2 data was reproduced from Willars *et al.,* [49]. (E): ATP-induced transient increase in cytosolic Ca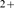 following P2X receptor activation in P2X3-transfected GT1 cells. Experimental data reproduced from He *et al.,* [56]. (F): ATP-induced transient increase in cytosolic Ca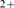 following P2Y receptor activation in Neuro2a cells. Experimental data reproduced from Lakshmi *et al.,* [48]. (G): ATP-dose dependent fraction of gated P2X3 channels for control (black) and cells treated with GDP-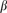-S (blue) from rat DRG neurons. Experimental data reproduced from Gerevich *et al.,* [31]. (H): UTP-dose dependent increases in peak cytosolic Ca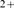 levels in rat DRG neurons. Experimental data was reproduced from Sanada *et al.,* [24].

The IP3/IP3R module recapitulated the steady-state regulation of IP3R channels as a function of IP3 and cytosolic calcium. IP3R receptors have previously been modeled as multimeric proteins composed of four identical subunits [40]–[45]. Single IP3R channel recordings have shown four conductance levels where one conductance level was correlated with greater opening time [46]. Based on these findings, we assumed that each IP3R had one IP3 and three calcium ion binding sites. Using this model, IP3R opening required sequential binding of IP3 and one calcium ion. We assumed IP3 binding induced an IP3R conformational change that blocked additional IP3 binding and exposed three calcium binding sites. Cytosolic calcium binding to the IP3-IP3R complex was assumed to initially open the IP3-IP3R channel allowing calcium transport from the ER to the cytosol. However, binding of a second or third calcium ion was assumed to downregulate the transport activity of the channel. Parameters and initial conditions for the IP3R channel model were estimated from independent steady-state measurements of the fraction of open IP3R channels as a function of cytosolic calcium and IP3 concentrations [46], [47]. The IP3R model reproduced steady-state channel behavior with a bell-shaped calcium dependency (Fig. 3A). The IP3R model also reproduced the fraction of open IP3R channels as a function of IP3 at a fixed calcium level (Fig. 3B). The ensemble of IP3R models reproduced between 73%–82% of the measured values within a single ensemble standard deviation and 100% of the measurements at three ensemble standard deviations.

The P2Y and PI modules recapitulated time-dependent cytosolic calcium and phosphoinositide measurements following ATP and UTP stimulation. The P2Y module was adapted from the Gq-protein coupled receptor (GPCR) and PLC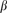 activation models of Bhalla *et al.* [32]. P2Y parameter values were constrained using two independent sets of time-resolved cytosolic calcium measurements following P2Y2 activation in Neuro2a cells and rat DRG neurons [24], [48]. To make sure the calcium dynamics were attributable solely to P2Y stimulation, we selected calcium measurements induced by ATP in the absence of extracellular calcium [48]. To capture dose-dependence and possible saturation effects, we used dose-dependent UTP-evoked calcium dynamics to constrain the P2Y module [24]. The model ensemble reproduced both ATP-P2Y2-evoked calcium dynamics and UTP-P2Y2-evoked calcium peak measurements (Fig. 3F and 3H). The P2Y module captured 75%–82% of the cytosolic calcium measurements within a single ensemble standard deviation and 100% of the measurements at three standard deviations. To capture the integration of PI metabolism with P2Y-driven calcium release, we used dynamic measurements of PIPx levels in stimulated SH-SY5Y human neuroblastoma cells to constrain the PI module [49]. Previous models have neglected PI recycling. Typically, these models assumed that PIP2 replenishment and IP3 degradation were constant or were mediated by enzymes with time-invariant activity [32], [42], [50], [51]. We addressed this issue by modifying a model of P2Y1-evoked calcium dynamics in platelets developed by Purvis *et al.* [34] by adding more phosphatases and kinase activities (Table 1). Following agonist stimulation, the concentration of both PIP (Fig. 3C) and PIP2 (Fig. 3D) decreased to approximately 30% of the basal level and then recovered albeit with different recovery rates. The model captured 50%–80% of the PIPx measurements within one ensemble standard deviation and between 80%–90% at three ensemble standard deviations. The agreement between measured and simulated PIP2 levels in particular was qualitatively correct but missing fine measurement features.

**Figure 4:**
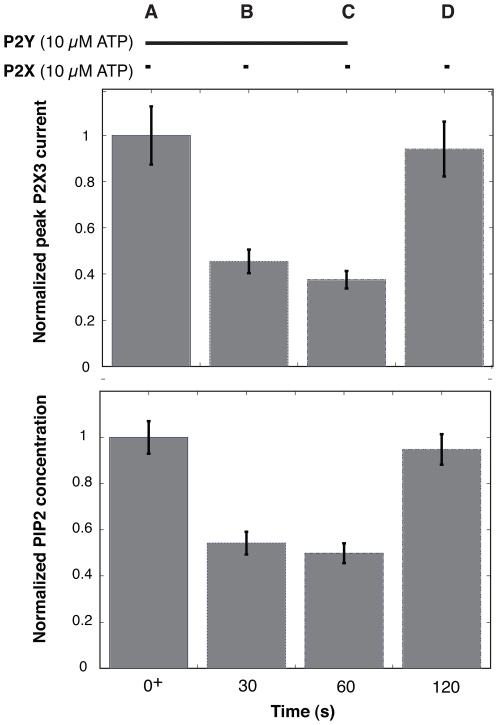
Fraction of gated P2X3 channels (top) and PIP2 levels (bottom) as a function of time and P2Y activation following P2X and P2Y activation with 10 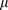
M ATP. The height of each bar denotes the ensemble mean while the error bars denote the standard error computed over the ensemble. A: Directly following the addition of ATP, the fraction of gated P2X3 channels and PIP2 levels are at a maximum despite P2Y activation. (B,C): The levels of gated P2X3 channels and PIP2 at 30 s (B) and 60 s (C) decreased relative to the wild-type. D: Gated P2X3 channels and PIP2 levels 60 s after the cessation of P2Y activation relax to their initial levels.

The P2X module recapitulated time-dependent cytosolic calcium measurements and the role of PI metabolism on P2X activity as a function of extracellular ATP stimulation. The structure of the P2X module was based on the study of Sokolova *et al.* [33]. Sokolova *et al.* experimentally and computationally explored the electrophysiological properties of P2X3 receptors using cultured rat sensory neurons. We modified the Sokolova model to reflect experimental evidence [52]–[55] suggesting that PIP2 stabilizes open P2X conformations (Table 1). We assumed two PIP2 binding events were required to stabilize open P2X channels. ATP-induced intracellular calcium dynamics measured in GT1 cells transfected with rat P2X3 receptors were used to train the behavior of the P2X module [56]. However, the GT1 experiments were done at a single ATP concentration. To capture ATP dose effects and constrain the influence of PIP2 on P2X channels, simulations of the fraction of open P2X3 receptors were compared with nominal rat DRG neurons and neurons loaded with the Gq-protein inhibitor GDP-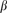-S as a function of ATP [31]. Consistent with Gerevich *et al.*, we assumed that P2X3-mediated current amplitude was proportional to the fraction of gated P2X3 channels [31]. The parameter ensemble captured the calcium dynamics following ATP-stimulation of transfected GT1 cells (Fig. 3E). The ensemble of models described 95% of the GT1 calcium measurements within one ensemble standard deviation. Using the hypothesis that PIP2 stabilized gated P2X3 receptors (Table 1), the model reproduced experimental observations in which ATP-induced peak current increased when GPCR activity was blocked by GDP-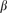-S (Fig. 3G). The model described 83%–100% of the measured peak current measurements as a function of ATP forcing within one ensemble standard deviation. We further explored the relationship between P2Y activation and the regulation of gated P2X channels by simulating simultaneous ATP-induced activation of P2X and P2Y receptors (Fig. 4). Directly following P2X/P2Y activation, there was no PIPx-mediated interaction between the receptors (Fig. 4A). However, when P2X activation was initiated 30 or 60 s after P2Y activation, the scaled peak current and PIP2 levels dropped to 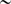
40% of the initial value (Fig. 4B and 4C). ATP-induced activation of P2X without P2Y stimulation (Gq cascade allowed to relax for 60 s) showed peak current levels approximately the same as the initial currents (Fig. 4D).

**Figure 5:**
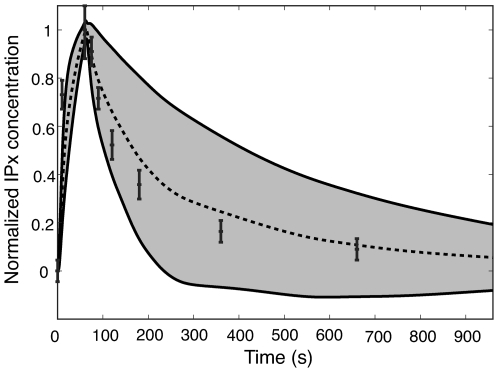
Predicted time course of total inositol phosphate levels (sum of IPx) versus experimental measurements in SH-SY5Y cells. The dashed line denotes the mean simulated value over the ensemble of model parameters while the shaded region denotes one ensemble standard deviation (N = 123). Experimental data are shown with error bars. The data was reproduced from Willars *et al.*, where muscarinic receptors (another class of G protein coupled receptor) was activated by carbachol in the human neuroblastoma cell line, SH-SY5Y [49]. Both the simulation and experiment were conducted with saturating levels of agonist. No parameters were adjusted for this comparison.

The model *predicted* inositol phosphate dynamics following G protein activation in SH-SY5Y human neuroblastoma cells. Phophoinositides such as PIP2 are precursors for inositol phosphates (IPx). Inositol phosphates carry out important regulatory functions, for example the regulation of IP3R channel activity. We tested the ability of the model to *predict* inositol phosphate dynamics given the PIPx training by comparing total inositol phosphate measurements (the sum of IPx) with model simulations following G-protein activation (Fig. 5). However, the agonist in these experiments was not ATP and the receptor was not a P2Y family member. Rather, IPx dynamics were measured following the activation of muscarinic receptors by carbachol. Muscarinic receptors are G protein-coupled acetylcholine receptors expressed on the surface of neurons [57]. We assumed that the G protein-coupled IPx dynamics from these receptors was similar to ATP stimulation of P2Y receptors. The model predicted 87% of the measured values (7 of 8 points) within one ensemble standard deviation and 100% of the values within three ensemble standard deviations.

### Sensitivity analysis suggested that cytosolic calcium homeostasis and phosphoinositide metabolism were important regardless of receptor activation

**Figure 6:**
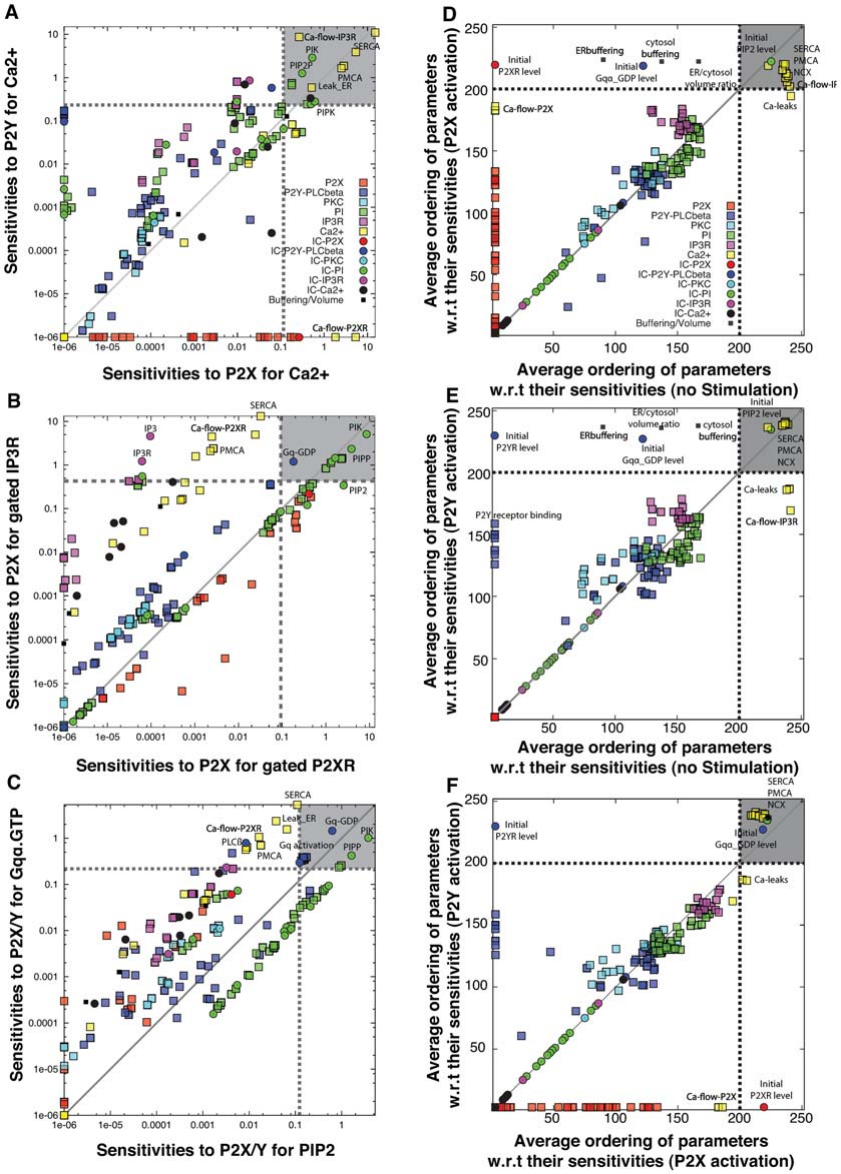
Sensitivity analysis as a function of model output and activation conditions. Squares denote rate constants while circles denote initial conditions organized by biological function. The mean values of the sensitivity coefficients calculated over the parameter ensemble are shown. Vertical and horizontal lines denote the top 10% of sensitive parameters or parameter combinations. Parameters in the shaded regions are highly sensitive regardless of conditions. A: Comparison of cytosolic calcium sensitivity for P2X versus P2Y activation (100
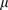
M ATP). B: Comparison of the sensitivity of gated P2X and IP3R channels for P2X receptor activation (100
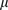
M ATP). C: Comparison of the sensitivity on PIP2 and Gq.GTP levels when both P2X and P2Y receptors were activated (100
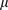
M ATP). (D,E,F): Average rank-ordering of parameter sensitivities as a function of receptor activation state.

To better understand the relative importance of network interactions on model outputs, we performed a sensitivity analysis over the parameter ensemble. Sensitivity analysis has been used previously to extract biological insight from signal transduction models despite model uncertainty [58]–[62]. Time averaged normalized sensitivities for three model outputs (cytosolic calcium, concentration of gated IP3R channels, Gq-protein activation) were computed over the parameter family as a function of P2Y and P2X activation (Fig. 6A–C). In addition, the coefficients of the eigenvector corresponding to the largest eigenvalue of the normalized sensitivity matrix product 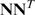 were used to analyze the effects of a combination of parameter changes on the whole system and rank order the model parameters with respect to their sensitivity [63] as a function of condition (Fig. 6D–F). The parameter ranking studies explored which combinations of parameters were globally important while the time averaged sensitivities looked only at specific model outputs. Dashed lines on each plot demarcate the upper 10% of the sensitive parameters for each condition. Sensitivity coefficients that lie along either axis denote parameters directly involved with particular activation states. Conversely, parameters that lie along the 45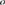 line in the upper 10% quadrant denote parameters which are important regardless of the activation state. Both rate constants and initial conditions were considered in the sensitivity analysis.

PI metabolism and the governance of cytosolic calcium levels were in the upper 10% of model parameters for both P2X and P2Y activation (Fig. 6A). When considering cytosolic calcium as the model output, interactions directly involved with P2X or P2Y activity segregated along their respective axis. The most sensitive parameters controlling the relationship between P2X and intracellular calcium was the permeability of P2XR channels with and without PIP2 stabilization. Conversely, ATP binding and PI recycling strongly influenced cytosolic calcium levels when only P2Y receptors were activated. Components involved in calcium homeostasis for example, SERCA and PMCA pumps were globally important for both P2X and P2Y activation. Similar results were obtained when looking at parameter groups for P2X activation (Fig. 6D) or P2Y activation (Fig. 6E). The combination studies supported the hypothesis that calcium homeostasis was critical (including IP3R channel regulation), with PI metabolism being secondarily important globally. From the simulation studies and the P2X sensitivity results, we expect phosphoinositides may regulate P2X channel activity. We explored which parameters regulated the activity of gated P2X versus IP3R channels when only P2X channels were active (Fig. 6B). G protein activation (PLC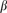, Gq-GDP), ion pumps and transporters were more important to the regulation of IP3R channels than to gated P2X receptors. However, parameters regulating PI metabolism and P2XR activation were important to both P2XR and IP3R activity. This suggested a cascade where PI metabolism influenced both IP3R and P2XR activity and IP3R activity was coupled to P2XR through intracellular calcium feedback. In particular, gated IP3R channels were sensitive to interactions involved with the PIP2 stabilization of gated P2XR channels. However, gated P2X channels were also indirectly sensitive to calcium through ATP-independent G protein activation. The initial condition of Gq-GDP was in the upper 10% of sensitive mechanisms regulating gated P2XR and IP3R channels.

## Discussion

Intracellular calcium levels are important to neuronal functions such as transmitter release and membrane excitability [8]–[13], [64], [65] as well as to pain networks, including the Bradykinin, COX-2, prostaglandins, and Serotonin signaling networks [66]. Thus, understanding the regulation of cytosolic calcium levels following agonist stimulation could be important to the development of treatments for acute and chronic pain [65]. In this study, we modeled ATP-induced calcium dynamics mediated by the P2 family of surface receptors. The model described the dynamics of 90 proteins, protein complexes or ions connected by 162 interactions. A family of model parameters was estimated using nine experimental training sets compiled from different cell-lines and laboratories. We estimated the parameter family using a Multi-Objective Thermal Ensemble (MOTE) technique. The MOTE algorithm identified parameter sets on or near the optimal trade-off surface between the individual training data constraints. The family of models simultaneously recapitulated the training data and predicted total inositol levels following GPCR activation. Sensitivity analysis was then used, over the family of parameter sets, to estimate which parameters were critical globally and key to specific model outputs (cytosolic calcium concentration, the fraction of gated IP3R channels and Gq-protein activation).

Phosphoinositide metabolism may mediate crosstalk between P2X and P2Y family members in neurons. Phosphoinositides, which are regulated by proteins with lipid recognition, kinase/phosphatase and phospholipase activities, have been suggested to control ion channel activity [67], [68]. Electrostatic interactions between the negatively charged headgroups of PIP2 and positively charged amino acids on the ion channels are thought to modulate the activity of the channels [55], [69]. For example, Zhao *et al.* showed that decreased PIP2 levels inhibited P2X3 currents in primary rat DRG neurons [52]. Bernier *et al.* showed that PIP2 modulated the current amplitude, recovery, and activation/de-activation kinetics of P2X1 channels in rat mesenteric arteries [53]. In this study, we hypothesized that plasma membrane phophoinositides modulated the activity of P2X channels by stabilizing the open conformation [52]–[55]. Using the hypothesized connectivity, the model explained the inhibitory effect of Gq-protein coupled P2Y receptor activity on P2X3 receptor-mediated currents in rat DRG neurons. It is widely accepted that P2X receptors, especially P2X3 and P2X2/3 selectively expressed in small DRG neurons, play an important role in pain transmission. However, the role of P2Y receptors in pain transmission remains unclear [30], [31], [70]–[73]. Metabotropic P2Y receptors, especially P2Y1 and P2Y2, are often co-expressed with P2X receptors in small DRG neurons and other cell types. Gerevich *et al.* suggested that negative synergy between stimulatory P2X3 and inhibitory P2Y receptors may be a novel regulatory mechanism to manage extreme pain signals [31]. The notion that P2Y receptors may dampen extreme P2X activity by modulating phosphoinositide levels was consistent with our simulation studies. Moreover, sensitivity analysis suggested that gated P2X channels could be strongly influenced by PI metabolism. However, the inverse relationship between P2Y and P2X activity may be cell-type or receptor subtype dependent. For example, P2X1 and P2Y receptors have been shown to have a positive synergy during platelet stimulation [74]. Moreover, inflammatory mediators like substance P and bradykinin, acting through PKC activity following Gq and phospholipase C cascades, potentiate currents through P2X3 and P2X2/3 channels in Xenopus oocytes [75]. Thus, more experimental and modeling studies are required to fully understand the physiological relationship between P2X and P2Y receptors in sensory neurons.

The rank-ordering of sensitive parameter combinations suggested the subsystems managing calcium homeostasis were structurally fragile. Evolutionarily optimized cellular infrastructure like ATPases, NCX or IP3R channels might be expected to be robust. However, these mechanisms were consistently ranked the most sensitive irrespective of receptor activation. PI metabolism was also predicted to be globally important regardless of which receptor was activated. Thus, malfunctions in or direct targeting of SERCA, PMCA or NCX channels may be more likely to elicit a global response independent of receptor activation or pain state. There are experimental studies which have tested or hypothesized the importance of these molecular components. For example, NCX inhibitors have been suggested for the treatment of cardiovascular disorders such as ischemia, arrhythmias and hypertension [76]. SERCA activity has been correlated with decreased neuronal viability in SH-SY5Y human neuroblastoma cells [77] and with the accumulation of ganglioside GM2 in the brain, a component of the neuropathology of Sandhoff's disease [78]. Abnormal SERCA or PMCA activities have been implicated in Multiple Sclerosis [79], hypertension [80], diabetes-induced disorders in nociceptive neurons [81], neuronal damage and death [79] and male infertility [82]. Thus, there is strong circumstantial evidence suggesting the maintenance of calcium homeostasis is a source of global fragility. However, targeting these globally important mechanisms in order to disrupt pain signals may not be appropriate and could perhaps initiate non-specific effects. Parameter ranking analysis also provided a means to differentiate between subsystems that were always sensitive versus those only sensitive following receptor activation. Mechanisms sensitive only after receptor activation could act as targets to manipulate the specific response of a neuron to stimulation. For example, P2X channel permeability or the initial levels of P2X receptors were sensitive only in the presence of ATP. This suggests that P2X channel inhibitors could selectively block different types of pain without intentionally interfering with other subsystems. P2X3 inhibitors have been explored clinically as novel analgesics [66], [83]. Research using selective P2X3 antagonists suggested these receptors were involved in both inflammatory and neuropathic pain [84]. However, this picture is complicated by a recent report showing P2X7 receptor activation down-regulated the expression of P2X3 in DRGs [85]. Thus, different receptor subtypes within the P2X family may be important in different types of pain. G protein cascade components were also only sensitive following P2Y activation. However, the value of P2Y and associated Gq-protein targets for the treatment of pain [72], [86] remains unclear as G protein agonists are likely to cross-react and interfere with non-pain networks.

## Materials and Methods

### Formulation and solution of the model equations

The calcium model was formulated as a set of coupled Ordinary Differential Equations (ODEs):
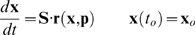
(1)


The symbol 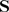 denotes the stoichiometric matrix (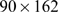). The quantity 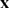 denotes the concentration vector of proteins, protein complexes or ions (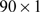). The term 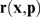 denotes the vector of reaction rates (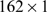). Each row in 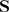 described a protein, protein complex or ion while each column described the stoichiometry of network interactions. Thus, the 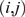 element of 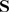, denoted by 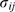, described how species 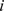 was involved in rate 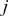. If 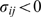, then protein 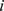 was consumed in 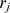. Conversely, if 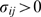, protein 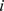 was produced by 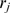. Lastly, if 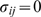, then protein 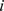 was not involved in process 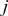.

We assumed mass-action kinetics for each interaction in the network. The rate expression for protein-protein interaction or catalytic reaction 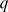:
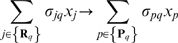
(2)was given by:
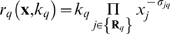
(3)


The set 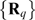 denotes reactants for reaction 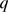. The quantity 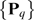 denotes the set of products for reaction 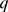. The 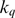 term denotes the rate constant governing the qth interaction. Lastly, 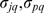 denote stoichiometric coefficients (elements of the matrix 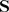). We treated every interaction in the model as non-negative. All reversible interactions were split into two irreversible steps. The mass-action formulation, while expanding the dimension of the P2 calcium model, regularized the mathematical structure. The regular structure allowed automatic generation of the model equations. In addition, an analytical Jacobian (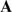) and matrix of partial derivatives of the mass balances with respect to the model parameters (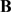) were also generated. Mass-action kinetics also regularized the model parameters. Unknown model parameters were one of only three types, association, dissociation or catalytic rate constants. Thus, although mass-action kinetics increased the number of parameters and species, they reduced the complexity of model analysis. The one exception to the mass-action formulation was the flow of ions through gated channels. We modeled this using a Nernst-like expression. Flow through gated channels from compartment 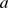 to 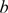 was assumed to be directly proportional to the fraction of open ion-channels 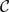 modified by the natural log of the concentration driving force between compartments:
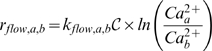
(4)where 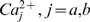 denotes the concentration of calcium in compartment j and 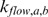 denotes the channel permeability. In this study, we did not consider intracellular concentration gradients. However, we accounted for extracellular, cytosolic and ER compartments by explicitly incorporating compartment specific species. We accounted for differences in the volume of each of the compartments using correction factors. The majority of the model equations were formulated based on the volume of the cytosol. ER species were derived from the cytosolic variants by dividing by 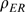 (the volume ratio of the ER and the cytosol) to correct for the different volume basis. We also corrected for the effect of Ca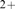 binding to protein buffers in both the cytosol and ER. At least 99% of Ca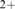 in the cytosol is bound to Ca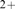 binding proteins of which there are about 200 encoded by the human genome [87], [88]. Similar to previous studies [89], we assumed that calcium buffering had sufficiently fast kinetics and the net effect of the buffers was to create effective volumes for the ER and the cytosol defined as 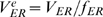 and 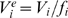 where 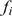 denoted the fraction of free calcium in the cytosol and 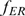 denoted the fraction of free calcium in the ER. The mass balance equations for Ca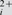 and Ca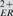 were multiplied by 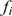 and 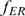 respectively. The values of 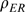, 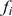, and 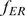 were estimated along with the other parameter values in the optimization framework.

The model equations were solved using the LSODE routine of the OCTAVE programming environment (http://www.octave.org; version 2.9.15) on an Apple Computer (Mac OSX; version 10.5.1, Cupertino CA). Model parameters and structure were taken from the literature or based on experimental data obtained in sensory neurons (see Table 1). Possible initial conditions were also taken from literature [32], [50]. However, the initial conditions of SERCA, PMCA, NCX were estimated as part of the parameter ensemble. In all simulations, we defined the homeostatic state as the stable equilibrium point in the absence of ATP stimulation.

### Sensitivity analysis of the model equations

Sensitivity values were computed by first calculating the first-order sensitivity coefficients at time 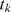:
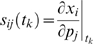
(5)which are solutions of the matrix differential equation:
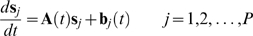
(6)subject to the initial condition 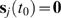. In Eqn. 6, the quantity 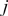 denotes the parameter index, 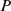 denotes the number of parameters in the model, 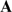 denotes the Jacobian matrix, and 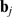 denotes the 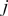 th column of the matrix of first-derivatives of the mass balances with respect to the parameter values (denoted by **B**). The Jacobian matrix and the matrix of first-derivatives of the mass balances w.r.t the parameter values are given by:
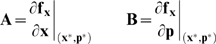
(7)where 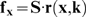 and 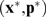 denotes a point along the system solution. Because the solution of the sensitivity equations required that we solve the model equations (to evaluate the 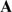 and 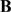 matrices), we formulated the sensitivity problem as an extended kinetic-sensitivity system of equations [90]

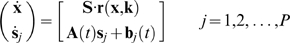
(8)where 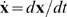 and 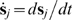. The model parameters were independent, thus we solved the extended kinetic-sensitivity system for multiple parameters in a single calculation using the LSODE routine of OCTAVE. The matrices 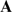 and 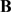 were estimated at each time step using their analytical expressions. The sensitivity coefficients were then normalized by the nominal parameter and state values:
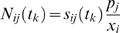
(9)


The normalized sensitivities could then by time-averaged by integration (Simpson's rule):
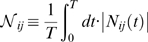
(10)


The normalized time-averaged sensitivity 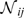 describes the time-averaged change in the state variable 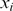 following a change in the parameter 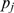. In addition to analyzing single sensitivity values, we used the Hearne method to find the most sensitive direction in the parameter space by estimating parameter combinations that maximized the difference in calcium model trajectories [63]. The absolute values of the eigenvector coefficients corresponding to the largest eigenvalue of the 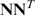 matrix were ranked-ordered for each parameter set and averaged over the ensemble.

### Estimation of the model parameter ensemble using a multi-objective thermal ensemble technique

**Figure 7:**
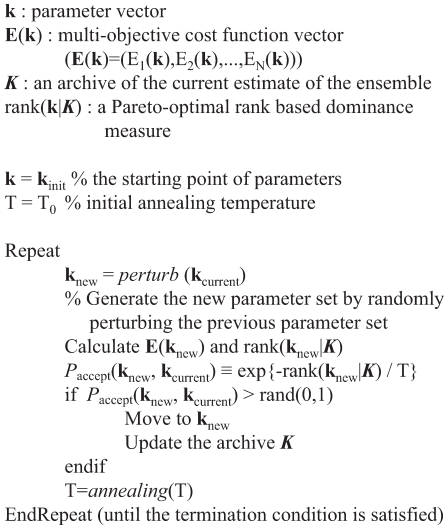
Multi-objective thermal ensemble algorithm used in this study.

The model parameters were estimated from nine independent data sets taken from multiple laboratories and cell-lines. We estimated an ensemble of model parameters from the training data using a Multi-Objective Thermal Ensemble (MOTE) method (Fig. 7). The MOTE algorithm integrated Simulated Annealing (SA) with Pareto optimality to estimate parameter sets on or near the optimal tradeoff surface between the distinct training sets. A Pareto-optimal energy function was constructed using rank-based fitness assignment. Denote a candidate parameter set generated at iteration 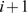 as 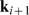. The Mean Squared Error (MSE) between simulations and the 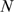 training sets at iteration 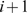 is given by:
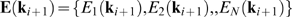
(11)where 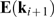 denotes the set of model simulation errors over all training data. The MOTE minimized the simulation error of each training constraint and balanced conflicts between constraints. We stored the parameter sets, model output and error estimates which lie along or near the trade-off surface through iteration 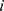 in the data structure 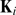. We computed the Pareto rank of 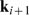 by comparing the simulation error at iteration 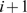 against the simulation archive 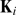. We used the Fonseca and Fleming scheme to compute the Pareto rank [91]. Suppose 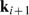 is worse in an Pareto-optimal sense than 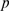 members in the current archive 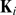, i.e., 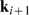 is dominated by 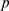 previous parameter sets. Then the Pareto rank of 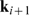 is given by:
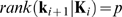
(12)


Parameter sets on the optimal trade-off surface have a rank equal to 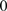 (no other current parameter sets are better). Sets with increasing non-zero rank are progressively farther away from the optimal trade-off surface. Thus, a parameter set with a 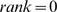 is *better* in a trade-off sense than 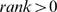. We used the Pareto rank to inform the SA calculation. The parameter set 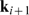 was accepted or rejected by the SA using the acceptance probability 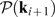:
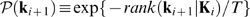
(13)where 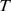 is the computational annealing temperature. As 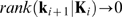, the acceptance probability moved toward one, ensuring that we explored parameter sets along the Pareto surface. Occasionally (depending upon 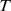) a parameter set with a high Pareto rank was accepted by the SA allowing a more diverse search of the parameter space. However, as 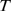 was reduced, the probability of this occurring decreased. Parameter sets could be accepted by the SA and 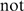 archived in 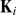. Only parameter sets with 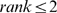 were included in 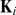 to ensure that we characterized the neighborhood near the trade-off surface. The parameter ensemble used in the simulation and sensitivity studies was generated from parameter sets in 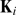.

## Supporting Information

Supplemental Materials S1 The archive for the Octave files for simulating the model
(0.02 MB ZIP)
